# Beyond the traditional oncology patient: the role of palliative care in patients with sickle cell disease receiving stem cell transplantation or gene therapy

**DOI:** 10.3389/fonc.2025.1535851

**Published:** 2025-02-13

**Authors:** Griffin S. Collins, Deena R. Levine, Alexis Leonard, Akshay Sharma, Liza-Marie Johnson

**Affiliations:** ^1^ Department of Oncology, Division of Quality of Life and Palliative Care, St. Jude Children’s Research Hospital, Memphis, TN, United States; ^2^ Department of Hematology, St. Jude Children’s Research Hospital, Memphis, TN, United States; ^3^ Department of Bone Marrow Transplantation and Cellular Therapy, St. Jude Children’s Research Hospital, Memphis, TN, United States; ^4^ Department of Oncology, The Bioethics Program, St. Jude Children’s Research Hospital, Memphis, TN, United States

**Keywords:** sickle cell disease, palliative care, hematopoietic stem cell transplantation, genetic therapies, health decision making, symptom management

## Abstract

People with severe sickle cell disease (SCD) are now presented with increasing access to curative-intent therapies including allogeneic hematopoietic stem cell transplantation (HCT) and gene therapy (GT). These high-risk, high-reward therapies offer hope for cure and prevention of further injury due to SCD, but they are toxic therapies that carry risk of additional morbidity and mortality. People with severe SCD suffer due to extreme pain and serious multi-system injury which is compounded by the effects of systemic racism. The increasing availability of these complex, sometimes novel, therapies with curative-intent highlights the role for specialist palliative care (PC) in the care of people with severe SCD. Multidisciplinary PC teams employ a holistic, person-centered approach to alleviating suffering by accompanying patients through high-stakes decision making, coping with life-threatening illness, and symptom management. The role for PC beginning early in HCT has been established, though PC is infrequently integrated in HCT. Little research exists regarding the role for PC in care of people with SCD. We present concepts of PC integration for people with SCD undergoing HCT or GT and advocate for PC integration beginning once patients consider a curative-intent therapy throughout the duration and following completion of treatment. As curative-intent therapies for patients with SCD continue to evolve, there is an opportunity for PC, HCT, and SCD teams to collaborate with patients to develop implementable models for high-quality, multidisciplinary care for people with severe SCD and their families.

## Introduction

Sickle cell disease (SCD) is an inherited blood disorder caused by a mutation that codes for an aberrant beta subunit of the hemoglobin molecule. The resultant sickle hemoglobin polymerizes under hypoxic conditions, deforming red blood cells and causing hemolysis, chronic ischemia, systemic inflammation, and multisystem organ damage (1, 2). In well-resourced settings, the manifestations can begin in infancy, however simple interventions such as newborn screening, immunizations, bacterial prophylaxis, and disease modifying therapies have improved pediatric life expectancy, changing SCD to a chronic disease of adulthood with substantial morbidity, poor quality of life (QOL), and a shortened lifespan. Tragically, in sub-Saharan Africa where the majority of children with SCD are born, a large number of children with SCD die before the age of 5 due to the lack of many of these simple interventions (3). SCD affects at least 100,000 people in the United States, an estimated 7.7 million individuals worldwide, and its prevalence is expected to dramatically increase in the coming decades (1, 4). SCD predominately affects people of African ancestry who have suffered historic and ongoing discrimination and systemic racism both inside and outside healthcare systems (5–7). Considering the growing prevalence and severity of the disease, there is a great need for curative therapies for SCD.

Palliative care (PC) is a multidisciplinary approach to care for people facing serious illness aimed at alleviating suffering and improving QOL. As part of the multidisciplinary team along with other medical and psychosocial-spiritual clinicians, PC specialists establish longitudinal relationships to accompany patients and their caregivers through serious illness regardless of prognosis or stage of illness (8, 9). While PC can be erroneously conflated with end-of-life care, PC teams are increasingly involved early in the care of people with serious illness to support them throughout their disease trajectory. Through skilled communication and relationship-based care PC specialists elicit a patient’s concept of their illness and its impact, as well as their goals and values, to ensure holistic, person-centered care. PC teams can assist patients with SCD in minimizing and alleviating suffering, support decision-making around therapeutic options, and support patients through intensive high-risk high-reward therapies with curative-intent.

### Treatments for sickle cell disease

Chronic transfusion therapy and medications such as hydroxyurea are disease-modifying treatments aimed at minimizing the symptoms of SCD. They may reduce the frequency of painful vaso-occlusive crises (VOC) and slow end-organ damage, but they are not curative, must be continued lifelong, are not universally effective, and may have serious side-effects (2, 10). Allogeneic hematopoietic stem cell transplant (HCT) and gene therapy (GT) are transformational, potentially curative treatments for patients with SCD (11, 12). HCT is an intensive therapy involving administration of a conditioning regimen with myeloablative and immunoablative chemotherapy and/or radiation followed by the infusion of a donor-derived hematopoietic stem cell product to replace the recipient’s hematopoietic and immune systems (13). HCT has been adapted to treat and cure a growing number of malignant and non-malignant disorders, including SCD (14). The first transplant for SCD occurred in 1983 when a girl with SCD who developed an acute leukemia underwent a successful myeloablative HCT from a human leukocyte antigen matched sibling donor (MSD) without SCD. When she recovered her hematopoietic system, she was cured of her leukemia and free from SCD as well. Since then, this therapy has been widely adopted. MSD HCT is the standard of care for patients with SCD seeking a cure (11, 15), however fewer than 15% of patients have an eligible MSD. Moreover, end organ damage limits those who can receive myeloablative conditioning, leaving many patients without curative treatment options. Allogeneic HCT modalities using alternative donors have historically led to high morbidity, graft failure, and mortality although recent advances in HCT conditioning regimens are significantly reducing these risks, increasing HCT availability for patients with severe SCD (11, 16).

As a monogenic disorder, autologous transplantation after genetic modification of patient-derived hematopoietic stem cells has long been envisioned with the hope to overcome the challenges of allogeneic HCT, namely the lack of available donors and the immunologic risks that accompany allogeneic transplantation (12). Clinical trials investigating GT for the treatment of SCD have been ongoing for nearly a decade, now with two GT products (lovotibeglogene autotemcel and exagamglogene autotemcel) commercially approved in the US in 2023 (17). GT for SCD includes mobilization of a patient’s own stem cells, ex vivo modification to correct for the abnormal hemoglobin gene and return of the genetically modified stem cells to the patient after administration of myeloablative chemotherapy (18). The therapy is promising, yet expensive, time intensive, and burdensome to patients who often require multiple cycles of mobilization and apheresis and is only accessible in high-resource settings (12, 19). **Table 1** compares HCT and GT in patients with SCD.

**Table 1:** Comparison of potentially curative therapies for sickle cell disease (20).

	Stem Cell Transplant	Gene Therapy
**Preferred Stem Cell Source**	HLA Matched Sibling Donor	Patient derived - cells are collected by apheresis, gene changes conducted ex vivo, and cells are reinfused into patient
**Alternative Stem Cell Sources**	Haploidentical (Half-Matched) Donor Matched Unrelated Donor Umbilical Cord	None. For some patients, collection of a sufficient number of stem cells will not be possible after multiple rounds of apheresis. Teams should manage patient expectations during preparation phase.
**Investigational Status**	Matched sibling donor HCT is no longer experimental HCT with alternative stem cell sources may be conducted in clinical trials	US FDA approved 2 therapies in 2023 Other investigational therapies are in development
**Duration of Therapy**	6-12 months after infusion	9-12 months prior to infusion 3-6 months after infusion
**Preparation for Therapy**	Central line placement	Central line placement
	Comprehensive organ function screening	Comprehensive organ function screening
	Exchange transfusions (may only require 1-2) (12)	Stop hydroxyurea for several months
	Therapy may be delayed by donor search	Monthly exchange transfusions (3-6 months) (12)
	Proceed once donor identified	Undergo multiple days of stem cell collection
		Several months wait for availability of stem cell product
**Typical Duration of Hospitalization**	4-6 weeks	4-6 weeks
**Chemotherapy**	Yes, 1–2-week conditioning regimen	Yes, about 1-week conditioning regimen
**Risk of Graft Rejection**	Yes, uncommon with newer regimens (21, 22)	Unlikely
**Risk of Graft Versus Host Disease**	Yes	No
**Immunocompromised**	Yes, due to conditioning chemotherapy and immunosuppression for GHVD prophylaxis	Yes, duration of neutropenia is less than with allo HCT. Lymphopenia is rare and immunological memory is usually not compromised.
**Risk of Infertility**	Yes, fertility preservation should be offered	Yes, fertility preservation should be offered
**Duration of Follow Up**	Several years	At least 15 years per US FDA guidance (23)

HLA, human leukocyte antigen; US FDA, United States Food and Drug Administration; GVHD, graft versus host disease.

### Integration of palliative care

Ample evidence demonstrates the benefit of integrating PC early in the disease course for people with serious illness (24–26). Early PC integration allows for development of a trusting therapeutic relationship, a foundational element of PC, which is especially crucial when working alongside members of minoritized communities who have endured discrimination.

**Figure 1:**
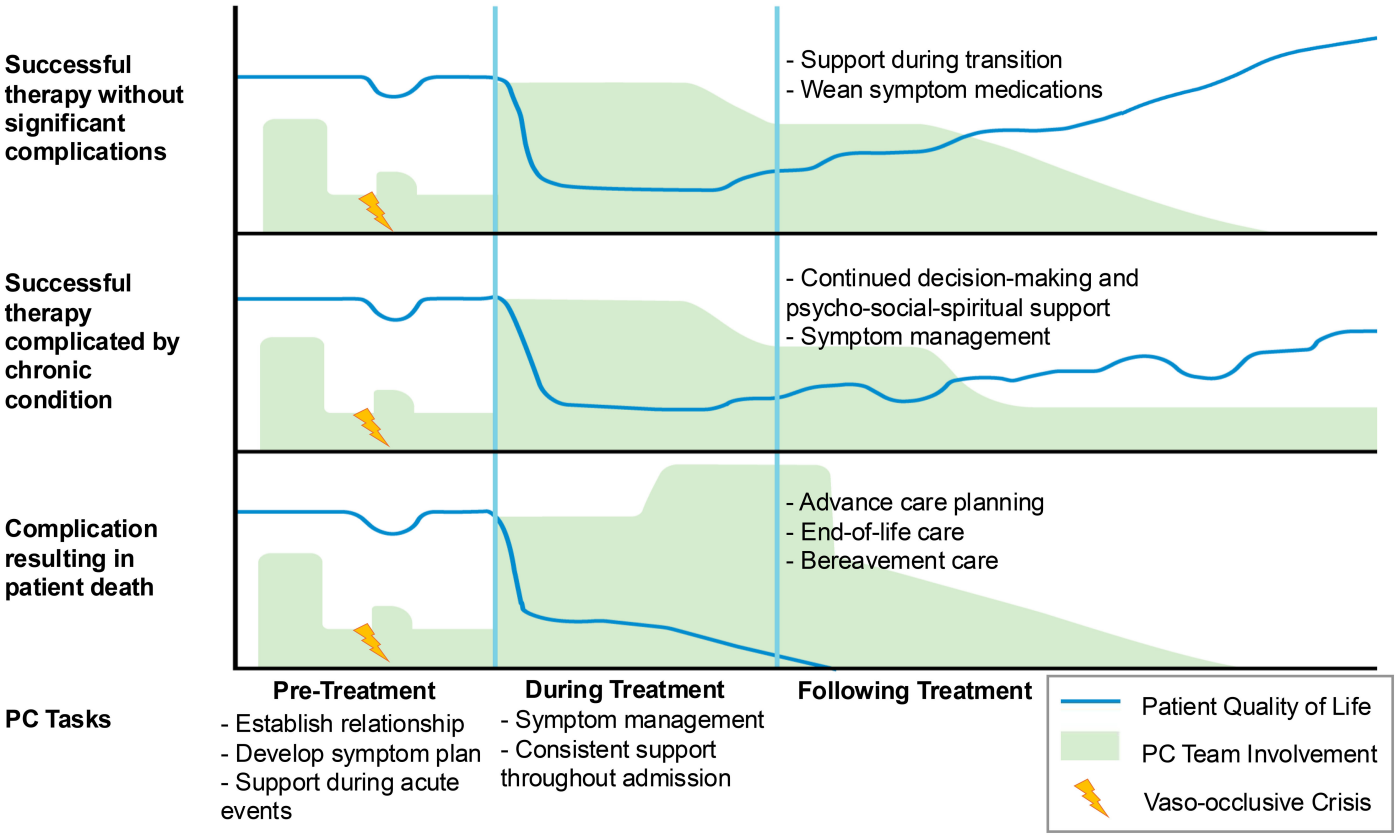
Conceptualization of PC involvements across three possible treatment trajectories.

Given the intensity, morbidity, and risk of mortality in HCT, some institutions have developed models for specialty PC integration to support patients and their caregivers throughout treatment (27–31). While patients eligible for HCT or GT for SCD have severe disease, the decision to pursue either treatment is unlike in malignant conditions treated with HCT where patients face an imminent risk of death without the therapy. Thus, the decision must be patient and family focused and made with the recognition that the risk and benefit ratio, optimal timing, and specific treatment options for any given patient are not fully known upfront. This presents an opportunity for PC teams to play a pivotal role for patients with SCD as HCT and GT become more readily available. **Figure 1** demonstrates how PC teams might engage with patients undergoing therapies with curative intent.

## Palliative care in the pre-treatment period

The role for PC involvement begins when patients first consider HCT or GT. The process to prepare a patient for these treatments can span months to years. During this time, patients and their families must make many important decisions while continuing to endure symptoms and suffering due to SCD.

### Shared-decision making

High-risk, low-certainty therapies require both informed consent and shared decision making to enhance the patient’s control over their medical care. Informed consent is a legal process whereas shared decision making is an ethical concept tailored to patient preference and values. PC specialists play an important role in the latter, supporting patients and their families as they consider the potential impact of their options. This support may be particularly helpful for patients from communities who have suffered discrimination in healthcare and medical research as they may have a well-earned mistrust of the healthcare system, especially when considering novel or complex therapies. PC teams have expertise in eliciting patients’ hopes and concerns and can help patients identify their priorities and goals, promoting self-advocacy, as they navigate uncertainty deciding whether to pursue curative-intent therapy.

Except for history of stroke, there are no universally agreed upon indications for HCT in SCD. While eligibility criteria for HCT and GT differ, they are generally reserved for patients with severe disease to justify the known risks and uncertainties (12, 21) although SCT may be offered to some patients with a lower disease burden who has an HLA-matched sibling donor given significant experience and known outcomes. Thus, any patient seriously considering HCT or GT likely has suffered considerable disease-related morbidity and increased mortality risk. For patients with cancer, HCT may represent the ultimate curative option for otherwise terminal diseases, and toxicity of treatment is accepted in hope for a cure. For patients with SCD who are not imminently dying of their disease, the decision-making calculus is different. Patients and their caregivers must decide when and if to “put their life on hold”—and rarely, if ever, in “imminent danger”—and agree to endure toxic therapy with hope of improving their QOL and halting a slowly progressive disease. Improved outcomes for younger patients compounds this difficult decision to proceed with a potentially life-threatening therapy when a child is doing well as opposed to waiting for more disease complications (11, 12, 32). Beyond general apprehension of undergoing curative-intent therapy, patients and families express specific concerns: effects of chemotherapy, cancer risk, and infertility (33, 34). PC teams trained in holistic care can accompany patients along their decision-making process as they balance their worries and hopes for these therapies.

Once a decision to pursue HCT or GT has been made, certain considerations and support are needed. When pursuing GT, patients must undergo stem cell collection through apheresis. Apheresis can be burdensome, and patients might have to undergo multiple procedures to collect sufficient stem cells to produce a GT product (35, 36). Conditioning chemotherapy for HCT and GT imparts a high risk of infertility. All patients who undergo these treatments should be offered fertility preservation (37). Patients must be counseled that HCT and GT only alter hematopoietic cells and the risk of their offspring inheriting SCD remains unchanged. Patients and families require transparent education and shared decision-making support as they consider the risks of fertility preservation, especially for female patients with SCD who undergo surgical fertility-preserving procedures (38).

### Psychosocial support

From birth, many patients with SCD receive care at centers managed by SCD specialty teams (21, 31). Psychosocial clinicians have integral roles in these teams caring for patients facing a life-long serious illness. When patients consider curative-intent therapy, their care is often transferred to HCT teams at specialized centers which may be different from where they were previously treated. This transition can be destabilizing for patients, and teams must be prepared to support patients as they adapt to a new setting. PC consultation at the outset of the HCT process establishes PC clinicians as trusted, consistent members of their team who understand the disease and can support them throughout their treatment.

### Symptom management

The hallmark of SCD is episodic, debilitating, and painful VOCs (1, 2). As patients undergo preparation for HCT or GT, their teams must be prepared to manage VOCs when they arise. Stem cell collection may precipitate VOCs for patients pursuing GT (39). Patients deserve care teams that are familiar with SCD, trust their report, and respond to their needs. Transplant teams that are new to the patient and family must be knowledgeable about managing SCD-related pain and should involve clinicians with this expertise including SCD, pain, and PC specialists.

Patients with severe SCD are likely to have repeated exposure to opioids. They may have developed opioid tolerance and require higher doses than opioid naïve patients for adequate analgesia. They may also report that certain opioids are more effective and others have unacceptable side effects. Many patients with severe SCD suffer from chronic pain. Clinicians should have a low threshold to evaluate for neuropathic pain and to assess for central sensitization for patients with chronic opioid exposure. With their expertise in symptom management and patient centered care, PC teams can work with patients to develop individualized pain plans prior to admission for HCT and remain involved to adapt them as needed. **Table 2** provides an overview of some approaches that can be used for pain management in SCD. Furthermore, the process of GT from consent to transplant is long, sometimes up to a year in length. As a chronic disease, management of sickle-related complications and re-evaluation of suitability of transplantation from SCD experts with support from PC teams is imperative throughout this treatment course.

**Table 2:** Selected pain management strategies for people with severe sickle cell disease*.

Selected Medications (40)					
Class	Medication	Common Route	Starting Dose		Considerations
			Under 50kg	50kg or Greater	
**Non-Opioid Medications**	Acetaminophen	PO	15mg/kg q6h	1000mg q6h	Avoid in patients with liver injury or risk of liver injury
	Ketorolac	IV	0.5mg/kg q8h	15mg q8h	Assess renal function prior to use; use with caution due to risk of nephrotoxicity.
	Ibuprofen	PO	10mg/kg q6h	600mg q6h	
	Celecoxib	PO	2mg/kg q12h	100mg q12h	
**Opioid Medications**	Morphine	PO	0.3mg/kg q4h	15mg q4h	Opioid medications are used for severe pain in addition to non-opioid medications Patients may have opioid tolerance due to prior exposure requiring higher doses for effective pain relief. Extended-release formulations available for some opioids.
		IV	0.1mg/kg q4h	5mg q4h	
	Hydromorphone	PO	0.08mg/kg q3h	2-4mg q3h	
		IV	0.015mg/kg q3h	0.2-0.6mg q3h	
	Oxycodone	PO	0.2mg/kg q4h	5-10mg q4h	
	Fentanyl	IV	1mcg/kg q30min	50mcg q30min	
		IN	1.5mcg/kg q30 min	100mcg q30min	Consider for patients without long term vascular access; intranasal can also provide rapid relief for patients (41). Short half-life.
		Patch	Fentanyl patches are for patients receiving medium- or long-term opioid therapy equivalent to at least 25mcg/hr fentanyl. Consult an opioid conversion tool for dosing guidance.		
	Methadone (42)	PO/IV	Useful for patients with concurrent opioid use disorder, dependence, or tolerance. Both methadone and buprenorphine require close monitoring and care with initiation, consult a specialist with experience prior to use.		
	Buprenorphine (43, 44)	PO/SL/Patch			
**Adjunct Medications**	Gabapentin	PO	5mg/kg nightly	300mg nightly	Clinicians should have a low threshold to consider neuropathic pain (45). Titrate slowly to limit drowsiness
	Ketamine (46)	IV	Useful as opioid sparing agents for patients with severe, refractory pain and for treating central sensitization and opioid hyperalgesia. Dosing is complex for these medications, consult a specialist with experience prior to use. Mexiletine can be helpful as maintenance therapy for patients with a good response to lidocaine infusions (47).		
	Lidocaine (46, 48)	IV			
	Mexiletine	PO			
Non-pharmacologic and Integrative Approaches					
Treatment	Considerations				
Hydration	Avoid hypertonic fluids and excess hydration, maintain euvolemia				
Heat	Heating pads, warm baths				
Distraction	Music, games, electronic devices, virtual reality				
Guided Imagery	These interventions should be provided by clinicians with appropriate training.				
Hypnosis					
Acupoint Therapy					
Massage					
Physical Therapy					
Strategies					
Standardization	Consistent approach to promote safe opioid use				
Individualization	Frequent reassessment and adaption to ensure patients receive safe and effective pain relief			

PO, oral; IV, intravenous; IN, intranasal; SL, sublingual.

*The medications, approaches, and strategies included here are intended as suggestions for treatment and not a comprehensive list.

### Risk of secondary malignancies

Patients who undergo HCT or GT face a lifelong risk of developing a secondary malignancy, most commonly acute myeloid leukemia. The lifetime risk of secondary malignancy is 1-3% for patients who undergo HCT for SCD (49). While the risk for GT is not fully known, the FDA has issued a boxed warning for one GT product regarding this risk, and the risk may be present for the entire class of ex vivo genetically modified autologous therapies (12). While few patients will develop a secondary malignancy, the knowledge of that risk can be distressing. Further, those who do suffer this complication will be facing a new serious illness requiring intense therapy which was caused by the treatment for a prior serious illness. PC teams can accompany patients as they weigh this risk with the benefits of undergoing HCT or GT and help them consider the consequences of their options.

## Palliative care during treatment

The role for the PC clinician continues once a patient is admitted for HCT or GT. Patients will suffer from unfamiliar symptoms, experience isolation, and may face unanticipated decisions during this arduous process.

### Symptom management

Unlike patients undergoing HCT for malignancies, patients with SCD are unlikely to have received chemotherapy prior to undergoing HCT or GT. This reality presents an opportunity for integrated PC teams to work alongside HCT and psychosocial-spiritual colleagues to anticipate and rapidly respond to suffering. Even with reduced intensity conditioning regimens, both the short- and long-term side-effects of chemotherapy can be distressing for patients with SCD. Hair loss can damage a patient’s body image and diminish their sense of self and identity. Patients with SCD need attentive and creative teams to rapidly respond to chemotherapy induced nausea and vomiting, with which they would not have prior experience.

Mucositis is a common complication of HCT as myeloablative chemotherapy causes inflammation of mucosal membranes leading to painful ulcerations throughout the gut (50). HCT teams routinely manage mucositis related pain, however, patients with SCD may respond to pain and treatment differently. Patients with SCD should receive maximal prophylactic therapy to mitigate mucositis and have expert teams ready to comprehensively manage their pain and distress.

### Psychosocial support and advance care planning

The suffering during HCT extends beyond the physical symptoms which patients experience. Despite SCD patients experiencing numerous encounters with the healthcare system throughout their lives, few will have endured a weeks-long hospitalization and even fewer will have experienced the strict isolation restrictions in place due to the immunocompromised status of HCT patients. The mental toll during HCT is burdensome when a patient’s course goes as expected, however when a patient experiences excess morbidity or mortality, their suffering and distress increase. Particularly for patients with SCD who undergo HCT or GT as an elective procedure, patients and caregivers could experience significant decisional regret. Patients may face unanticipated complications during HCT or GT including treatment related toxicity, graft failure which may necessitate an urgent HCT from a new donor (21), and/or life-threatening organ damage. Conditioning chemotherapy is immunosuppressive and toxic, putting patients at risk of life-threatening infections and injury to multiple organs. When PC teams are integrated early in care, patients and families can develop trusting, therapeutic relationships with these teams which enable and facilitate high-stakes conversations if patients suffer life-threatening consequences that lead to changes in goals of care. PC teams work to ensure that patients receive care that is aligned with their goals throughout all phases of illness and treatment and play a pivotal role in providing compassionate care that ensures comfort and protects dignity for patients at the end of life (31).

## Palliative care following treatment

PC teams can offer support for patients as they adjust to life after undergoing transformational therapy. Following discharge, patients may encounter new challenges related to their treatment and some will continue to experience chronic symptoms related to SCD.

### Burden of care

Immediately after completion of HCT or GT, patients remain immunocompromised for a period of weeks to months. Due to the risk of severe infection, patients are advised to isolate and avoid crowded places. Patients typically have many prescriptions and frequent follow-up appointments. Even with appropriate anticipatory guidance, patients and their caregivers may be overwhelmed by the demands of their care after therapy. Additionally, patients may find that friends, family, and colleagues may not understand the burden of their care and not understand why they must remain isolated for a prolonged period of time.

### Graft versus host disease

HCT carries a risk of graft versus host disease (GVHD), where the transplanted immune system attacks the recipient. The risk of GHVD for recipients of an allogeneic HCT ranges from 20-40% depending upon the donor and the regimen and, unlike for patients who undergo HCT for hematologic malignancies, has no potential benefit (11, 21). GVHD severely impairs a patient’s QOL and is a major cause of HCT-related mortality (51–53).

### Chronic symptoms after treatment

Following HCT, most patients will experience a reduction in VOCs, normalization of cerebral blood flow and oxygen extraction, stabilization of pulmonary function, and improvement in cardiac and renal function (54). However, some patients will continue to experience symptoms following treatment. For unclear reasons, some patients will continue to endure painful events after HCT. Up to 40% of patients have required hospitalization for severe pain in the first year following HCT (55), though this risk is greatly reduced in the second year after HCT (56). As it is novel therapy, less is known about outcomes following GT, however a >90% reduction in pain events after GT has been reported (17, 57, 58). Chronic pain syndromes may persist and require ongoing treatment. And while cerebral hemodynamics improve, pre-existing neurological deficits will not be corrected by HCT or GT.

SCD-related pain is complex and sometimes difficult to differentiate from other causes of pain, including pain memory, opioid withdrawal, opioid induced hyperalgesia, or neuropathic pain. Integrated PC specialists can play a crucial role in supporting patients’ needs for ongoing symptom management following recovery from HCT or GT, understanding their goals for treatment, and supporting patients through the frustration of ongoing pain after being “cured” of SCD. This long-term, goal oriented, and collaborative approach to pain management can facilitate weaning opioids and other medications (59, 60).

### Identity following treatment

For many patients with SCD, the illness becomes an integral part of their identity with impacts on their relationships and communities. After undergoing HCT or GT, this aspect of their identity is irrevocably changed. Patients may wonder where they belong once they “no longer have” the chronic illness that shaped much of their life. Beyond this social suffering, some patients may experience survivor’s guilt that they no longer suffer from the disease that afflicts many of their peers. These feelings may be more profound for individuals from minoritized racial groups and groups with a communal sense of identity. PC specialists are uniquely positioned to accompany patients who experience seemingly conflicting emotions—like hope and worry, relief and grief—simultaneously.

## Discussion

Patients with severe SCD are presented with increased availability of treatments designed to cure SCD and prevent further suffering from the disease. However, these therapies are toxic and accompanied by serious risks. This high-stakes decision-making and treatment presents an opportunity for specialty PC teams to collaborate with SCD and HCT teams to improve the quality of life for people with severe SCD. There is little research regarding PC for people with SCD, and the emergence of these therapies presents an opportunity for patients and caregivers to engage with their teams to design effective models of PC integration.

### Barriers

Teams may encounter barriers to ensure that all patients with SCD considering potentially curative therapies receive specialty PC. PC team integration in HCT is variable across sites and, overall, rare. PC teams are even less integrated in hematology or SCD teams. Ample evidence demonstrates the benefits of early integration of PC in HCT (61, 62), thus it is critical that institutions invest in well-staffed multidisciplinary PC teams to meet the needs of their patients. While PC clinicians have the necessary skills and expertise to care for people with SCD, many PC clinicians have limited experience caring for this patient population and must adapt their skills to the unique goals and needs of people with SCD. To overcome potential stigma of PC, clinicians should emphasize PC’s expertise in symptom management, supporting patients with serious illness, and care coordination for all HCT and GT patients.

### Conclusion

PC teams are uniquely suited to meet the needs of patients with SCD undergoing curative-intent therapies through relationship-based care, pharmacologic and non-pharmacologic symptom management, skilled communication, and care coordination ensuring goal-concordant care. This developing field presents novel opportunities for HCT, SCD, and PC teams to develop collaborative models of care, offering hope to relieve the long-standing suffering caused by SCD.

## Data Availability

The original contributions presented in the study are included in the article/supplementary material. Further inquiries can be directed to the corresponding author.
